# Ciprofloxacin-Resistant *Salmonella enterica* Serotype Typhimurium, China

**DOI:** 10.3201/eid1403.070857

**Published:** 2008-03

**Authors:** Shenghui Cui, Jingyun Li, Ziyong Sun, Changqin Hu, Shaohong Jin, Yunchang Guo, Lu Ran, Yue Ma

**Affiliations:** * State Food and Drug Administration, Beijing, People’s Republic of China; † Huazhong University of Science and Technology, Wuhan, People’s Republic of China; ‡ Chinese Center for Disease Control and Prevention, Beijing, People’s Republic of China

**Keywords:** Salmonella Typhimurium, outpatients, ciprofloxacin, resistance, dispatch

## Abstract

We characterized 44 *Salmonella enterica* serotype Typhimurium isolates from Tongji Hospital outpatients in Wuhan, China, May 2002–October 2005. All 31 ciprofloxacin-resistant isolates were also resistant to >8 other antimicrobial drugs and carried >2 mutations in GyrA and 1 mutation in ParC. Class 1 integrons were identified in 37 isolates.

Salmonellae are a common cause of community-acquired foodborne bacterial gastroenteritis worldwide. The incidence of *Salmonella* infections in the People’s Republic of China has not been well documented. However, in the United States, ≈1.4 million persons are infected by *Salmonella* spp. each year (*1*). Although >2,500 serotypes have been reported, *Salmonella enterica* serotype Typhimurium is 1 of the leading serotypes causing salmonellosis worldwide (*2*). Fluoroquinolones such as ciprofloxacin are strongly recommended for treatment of severe *S.* Typhimurium infections in adults (*3*).

In this study, we characterized all *S.* Typhimurium isolates recovered from May 2002 through October 2005 from outpatients of Tongji Hospital, Wuhan, China, a sentinel hospital in the National Center for Surveillance of Antimicrobial Resistance. During the time of this study, Tongji Hospital strictly followed the recommendation for treatment of severe *S.* Typhimurium infections.

## The Study

We analyzed stool samples from outpatients who came to Tongji Hospital from the local community for treatment of diarrhea during the study period. A total of 44 *S.* Typhimurium isolates were recovered from the samples. *S.* Typhimurium was identified by using standard biochemical tests and commercial typing antiserum (Statens Serum Institute, Copenhagen, Denmark) according to the manufacturer’s instructions. MICs of 15 antimicrobial drugs (Table) were determined by using the broth-microdilution method; susceptibility to streptomycin was measured by using the disk-diffusion method as recommended by the Clinical and Laboratory Standards Institute (*4*). All isolates were further characterized by mutation analysis in the quinolone-resistance determining regions (QRDRs), pulsed-field gel electrophoresis (PFGE), and screening for class I integrons and β-lactamase genes as previously described (*5*–*8*).

**Table:** Resistance phenotypes of *Salmonella enterica* serotype Typhimurium  isolated from Tongji Hospital outpatients, Wuhan, China, May 2002–October 2005

Antimicrobial agent	MIC, μg/mL*	No. resistant isolates (n = 44)
Phenicols (chloramphenicol)	>32	33
Penicillins		
Ampicillin	>32	35
Amoxicillin–clavulanic acid	>32/16	32
Cephalosporins		
Cefepime	>32	0
Cefotaxime	>64	0
Ceftriaxone	>64	0
Tetracyclines (tetracycline)	>16	36
Aminoglycosides		
Amikacin	>64	2
Gentamicin	>16	35
Kanamycin	>64	15
Streptomycin†	NA	40
Sulfonamides and potentiated sulfonamides		
Sulfamethoxazole	>512	39
Trimethoprim-sulfamethoxazole	>4/76	36
Quinolones and fluoroquinolones		
Nalidixic acid	>32	36
Ciprofloxacin	>4	31

*MICs were determined by the broth-microdilution method; results were interpreted in accordance with the interpretive standards of the Clinical and Laboratory Standards Institute (*4*). †Resistance to streptomycin was determined by disk-diffusion method. NA, not applied.

Of the 44 isolates, 36 (82%) were resistant to nalidixic acid and 31 (70%) were resistant to ciprofloxacin (Table). Only 3 isolates, recovered in 2002, were susceptible to all 15 tested antimicrobial drugs; 36 (82%) displayed resistance to at least 8 drugs. Of 13 antimicrobial drug–resistant phenotypes identified, the most often observed phenotype (21/44) was resistance to amoxicillin–clavulanic acid, ampicillin, chloramphenicol, ciprofloxacin, gentamicin, nalidixic acid, sulfamethoxazole, streptomycin, trimethoprim–sulfamethoxazole, and tetracycline (R-type AcAmCCpGNSStSxtT). All isolates were susceptible to cefotaxime and ceftazidime; 5 isolates obtained in 2004 were intermediately susceptible to cefepime (MIC 16 μg/mL) (Appendix Figure).

Overall, 8 PFGE strain types (A–H) and 6 clusters (1–6) were identified. All isolates that belonged to clusters 1, 2, and 4 were resistant to ciprofloxacin and to 8–11 other antimicrobial drugs. Two dominant patterns, B and F, were identified and included 16 and 10 ciprofloxacin-resistant isolates, respectively. Among 16 isolates of pattern B, 14 isolates showed the R-type AcAmCCpGNSStSxtT, and 1 was additionally resistant to kanamycin. In pattern F, 4 isolates showed the R-type AcAmCCpGNSStSxtT, and 5 were additionally resistant to kanamycin.

Point mutations in the QRDR of *gyrA*, *parC,* or *parE* were identified in 35 of 36 nalidixic acid–resistant isolates, whereas no *gyrB* mutations and no *qnr* plasmid were found. For 5 nalidixic acid–resistant and ciprofloxacin low-level–resistant isolates, 4 isolates harbored single (D87N) or double (S83F, D87N) mutations in GyrA, and no mutation was found in 1 isolate (ST6). All 31 ciprofloxacin-resistant isolates accumulated a minimum of 3 mutations: GyrA(S83F, D87N), ParC(S80R) (28 isolates) or GyrA(S83F, D87G), ParC(S80R) (3 isolates). Two ciprofloxacin-resistant isolates with PFGE pattern C and 1 isolate with PFGE pattern A2 harbored an additional mutation in ParE (S458P) (Appendix Figure).

Of 39 sulfamethoxazole-resistant isolates encompassing PFGE clusters 1, 2, 3, and 4, 37 possessed class 1 integrons. All class 1 integron–positive isolates were resistant to 6–12 antimicrobial drugs; 2 distinct class 1 integrons were identified in 37 isolates. Of isolates obtained from 2002 through 2005, 32 contained a 1.9-kb integron gene cassette *dhfrXII-orfF-aadA2*. In 2004 and 2005, 3 and 2 isolates, respectively, contained a 2-kb integron gene cassette *bla*_OXA-30_-*aadA1*. None of the 36 ampicillin-resistant isolates contained TEM or SHV enzyme, but OXA-30 gene was detected in 32 isolates, identical in DNA sequence to GenBank AF255921. All 32 isolates harboring OXA-30 enzyme showed MICs to cefepime of 2–16 μg/mL, whereas isolates lacking OXA-30 showed MICs to cefepime of <1 μg/mL. In 2004, 5 isolates harboring OXA-30 enzyme with PFGE pattern F showed intermediate susceptibility to cefepime. All ciprofloxacin-resistant *S.* Typhimurium isolates also harbored class 1 integron, β-lactamases, and were phenotypically resistant to 8–11 additional antimicrobial drugs (Appendix Figure).

## Conclusions

We report a high incidence of fluoroquinolone-resistant *S.* Typhimurium isolates from Tongji Hospital outpatients. The MIC variation for ciprofloxacin differed 2- to 4-fold in isolates that had the same QRDR mutation profile, which implies that other mechanisms might partially contribute to the resistance phenotype (Appendix Figure). After PFGE analysis, *S.* Typhimurium isolates were grouped into 3 ciprofloxacin-susceptible clusters and ciprofloxacin-resistant clusters. Similar distribution patterns have also been observed in isolates from Japan (*9*), which suggests a distinct genetic lineage for ciprofloxacin-resistant isolates that have become dominant. Studies have reported that ciprofloxacin-resistant *S.* Typhimurium isolates were usually resistant to multiple drugs (*9*,*10*). In this study, all ciprofloxacin-resistant *S.* Typhimurium isolates were resistant to 8–11 additional antimicrobial drugs. Among the 32 isolates harboring OXA-30 enzyme in this study, only 5 with PFGE pattern F showed intermediate resistance to cefepime, which suggests different levels of OXA gene expression or the contribution of other unknown mechanisms.

The high incidence of quinonlone-resistant *S.* Typhimurium isolates in this study might be affected by several factors. First, patients infected by antimicrobial drug–resistant *S.* Typhimurium strains had higher rates of hospitalization than did patients infected by susceptible strains (*11*,*12*), and the isolates in this study were from a university-affiliated medical center that usually treats patients with severe illness. Second, US studies have estimated that half of outpatient antimicrobial drugs were inappropriately prescribed for conditions such as viral illness (*13*). In China, inappropriate prescriptions might be even more common because antimicrobial drug prescriptions in hospitals are a source of profit. Although we do not have patient antimicrobial drug–use information, the easy access to antimicrobial drugs raises the possibility that outpatients might have taken fluoroquinolones after the onset of the illness but before the collection of stool specimens. Third, because livestock products are a common source of salmonellosis, the dissemination of ciprofloxacin-resistant *S.* Typhimurium might have been facilitated by the use of fluoroquinolones in livestock production (*2*). Last, use of other antimicrobial drugs, such as ampicillin, gentamicin, or streptomycin, may also contribute to the spreading of fluoroquinolone-resistant *S.* Typhimurium because all the ciprofloxacin-resistant isolates were also resistant to 8–11 additional antimicrobial drugs.

Although fluoroquinolone-resistant isolates were prevalent in Tongji Hospital, ciprofloxacin is still empirically used to treat salmonellosis in adults, due partly to the absence of systematic surveillance programs to actively monitor antimicrobial drug resistance in *Salmonella* spp. Because local data on antimicrobial drug susceptibility are less available, we strongly recommend that hospitals and national and local health laboratories develop and maintain the capacity to perform *Salmonella* culture and in vitro susceptibility testing.

## Supplementary Material

Appendix Figure Dendrogram of patterns obtained by pulsed-field gel electrophoresis of Salmonella enterica serotype Typhimurium isolates from outpatients, Wuhan, People's Republic of China. Statistical analysis of restriction patterns was performed by using BioNumerics software (Applied Maths, St-Martens-Latern, Belgium) with the Dice similarity coefficient. The tree indicating relative genetic similarity was constructed on the basis of the unweighted pair-group method of averages, position tolerance of 0.5%. Values of 100% mean that the strains are identical. To be considered part of a cluster, the DNA patterns could not differ from each other by >10%. Similarity that differed by <5% was considered to represent subtypes within the main group (e.g., A1, A2, and A3). *Isolates resistant to ampicillin were screened for the ß-lactamase gene blaOXA-30; NT, not tested. †Sulfamethoxazole-resistant isolates were screened for class 1 integrons, and the following genes were identified: the gene for aminoglycoside adenyltransferase (aadA2), the gene for dihydrofolate reductase (dhfrXII), the ß-lactamase gene (blaOXA-30), and an open reading frame (ORF). PFGE, pulsed-field gel electrophoresis; N, nalidixic acid; CIP, ciprofloxacin; QRDRs, quinolone resistance–determining regions; FEP, cefepime; Ac, amoxicillin/clavulanic acid; Am, ampicillin; C, chloramphenicol; Cp, ciprofloxacin; G, gentamicin; S, sulfamethoxazole; St, streptomycin; Sxt, trimethoprim/sulfamethoxazole; T, tetracycline; Ak, amikacin; NT, not tested; K, kanamycin; –, susceptible to all tested antimicrobial drugs.
