# Supplementary figures and images for: Ciprofloxacin-Resistant Salmonella enterica Serotype Typhimurium, China
Source: Emerg Infect Dis. 2008 Mar;14(3):493–5. doi: 10.3201/eid1403.070857 (PMC2570801; doi:10.3201/eid1403.070857)

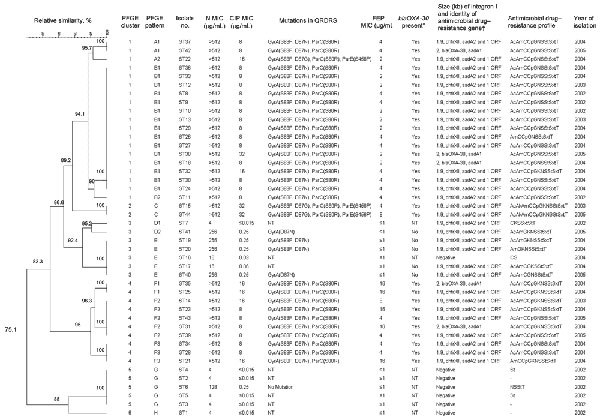

Supplement: Appendix Figure — Dendrogram of patterns obtained by pulsed-field gel electrophoresis of Salmonella enterica serotype Typhimurium isolates from outpatients, Wuhan, People's Republic of China. Statistical analysis of restriction patterns was performed by using BioNumerics software (Applied Maths, St-Martens-Latern, Belgium) with the Dice similarity coefficient. The tree indicating relative genetic similarity was constructed on the basis of the unweighted pair-group method of averages, position tolerance of 0.5%. Values of 100% mean that the strains are identical. To be considered part of a cluster, the DNA patterns could not differ from each other by >10%. Similarity that differed by <5% was considered to represent subtypes within the main group (e.g., A1, A2, and A3). *Isolates resistant to ampicillin were screened for the ß-lactamase gene blaOXA-30; NT, not tested. †Sulfamethoxazole-resistant isolates were screened for class 1 integrons, and the following genes were identified: the gene for aminoglycoside adenyltransferase (aadA2), the gene for dihydrofolate reductase (dhfrXII), the ß-lactamase gene (blaOXA-30), and an open reading frame (ORF). PFGE, pulsed-field gel electrophoresis; N, nalidixic acid; CIP, ciprofloxacin; QRDRs, quinolone resistance–determining regions; FEP, cefepime; Ac, amoxicillin/clavulanic acid; Am, ampicillin; C, chloramphenicol; Cp, ciprofloxacin; G, gentamicin; S, sulfamethoxazole; St, streptomycin; Sxt, trimethoprim/sulfamethoxazole; T, tetracycline; Ak, amikacin; NT, not tested; K, kanamycin; –, susceptible to all tested antimicrobial drugs. [file 07-0857-app-s1.jpg]
